# Machine learning–based prediction of disease progression in primary progressive multiple sclerosis

**DOI:** 10.1093/braincomms/fcae427

**Published:** 2025-01-08

**Authors:** Michael Gurevich, Rina Zilkha-Falb, Jia Sherman, Maxime Usdin, Catarina Raposo, Licinio Craveiro, Polina Sonis, David Magalashvili, Shay Menascu, Mark Dolev, Anat Achiron

**Affiliations:** Multiple Sclerosis Center, Sheba Medical Center, Ramat-Gan 5262, Israel; Sackler School of Medicine, Tel-Aviv University, Tel Aviv 6139601, Israel; Multiple Sclerosis Center, Sheba Medical Center, Ramat-Gan 5262, Israel; Research & Development, Genentech, Inc., South San Francisco, CA 94080, USA; Research & Development, Genentech, Inc., South San Francisco, CA 94080, USA; Roche Innovation Center Basel, Hoffmann-La Roche Ltd., Basel 4070, Switzerland; Roche Innovation Center Basel, Hoffmann-La Roche Ltd., Basel 4070, Switzerland; Multiple Sclerosis Center, Sheba Medical Center, Ramat-Gan 5262, Israel; Multiple Sclerosis Center, Sheba Medical Center, Ramat-Gan 5262, Israel; Multiple Sclerosis Center, Sheba Medical Center, Ramat-Gan 5262, Israel; Sackler School of Medicine, Tel-Aviv University, Tel Aviv 6139601, Israel; Multiple Sclerosis Center, Sheba Medical Center, Ramat-Gan 5262, Israel; Sackler School of Medicine, Tel-Aviv University, Tel Aviv 6139601, Israel; Multiple Sclerosis Center, Sheba Medical Center, Ramat-Gan 5262, Israel; Sackler School of Medicine, Tel-Aviv University, Tel Aviv 6139601, Israel

**Keywords:** primary progressive multiple sclerosis, prediction of disability, gene expression

## Abstract

Primary progressive multiple sclerosis (PPMS) affects 10–15% of multiple sclerosis patients and presents significant variability in the rate of disability progression. Identifying key biological features and patients at higher risk for fast progression is crucial to develop and optimize treatment strategies. Peripheral blood cell transcriptome has the potential to provide valuable information to predict patients’ outcomes. In this study, we utilized a machine learning framework applied to the baseline blood transcriptional profiles and brain MRI radiological enumerations to develop prognostic models. These models aim to identify PPMS patients likely to experience significant disease progression and who could benefit from early treatment intervention. RNA-sequence analysis was performed on total RNA extracted from peripheral blood mononuclear cells of PPMS patients in the placebo arm of the ORATORIO clinical trial (NCT01412333), using Illumina NovaSeq S2. Cross-validation algorithms from Partek Genome Suite (www.partek.com) were applied to predict disability progression and brain volume loss over 120 weeks. For disability progression prediction, we analysed blood RNA samples from 135 PPMS patients (61 females and 74 males) with a mean ± standard error age of 44.0 ± 0.7 years, disease duration of 5.9 ± 0.32 years and a median baseline Expanded Disability Status Scale (EDSS) score of 4.3 (range 3.5–6.5). Over the 120-week study, 39.3% (53/135) of patients reached the disability progression end-point, with an average EDSS score increase of 1.3 ± 0.16. For brain volume loss prediction, blood RNA samples from 94 PPMS patients (41 females and 53 males), mean ± standard error age of 43.7 ± 0.7 years and a median baseline EDSS of 4.0 (range 3.0–6.5) were used. Sixty-seven per cent (63/94) experienced significant brain volume loss. For the prediction of disability progression, we developed a two-level procedure. In the first level, a 10-gene predictor achieved a classification accuracy of 70.9 ± 4.5% in identifying patients reaching the disability end-point within 120 weeks. In the second level, a four-gene classifier distinguished between fast and slow disability progression with a 506-day cut-off, achieving 74.1 ± 5.2% accuracy. For brain volume loss prediction, a 12-gene classifier reached an accuracy of 70.2 ± 6.7%, which improved to 74.1 ± 5.2% when combined with baseline brain MRI measurements. In conclusion, our study demonstrates that blood transcriptome data, alone or combined with baseline brain MRI metrics, can effectively predict disability progression and brain volume loss in PPMS patients.

## Teaser

Gurevich *et al.* report a novel machine learning approach that successfully predicted 120 weeks’ disability and brain atrophy in primary progressive multiple sclerosis patients. The innovative aspect lies in the integration of the high-throughput blood RNA-sequencing with brain radiological variables.

## Graphical Abstract

**Graphical Abstract:**
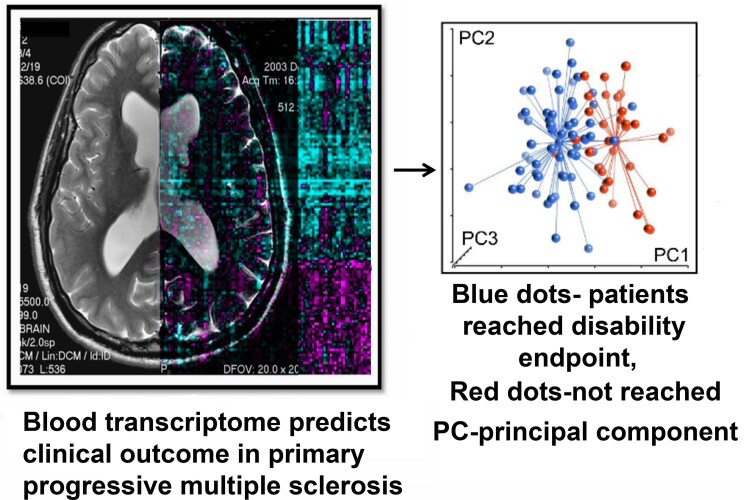

## Introduction

Primary progressive multiple sclerosis (PPMS) affects 10–15% of multiple sclerosis patients characterized by gradually worsening and accumulating disability.^1-3^ The clinical trajectory of PPMS varies significantly among patients.^4^ The disease can be radiologically active or inactive, either with or without clinical progression.^5^ In a recent study, we demonstrated that within 2 and 5 years of onset, 21.4 and 32.8% of PPMS patients, respectively, progress to moderate disability, while 3.3 and 14.1% progress to severe disability. In contrast, 4.7% do not progress to moderate disability even for 20 years.^6^ Treatment options for PPMS are limited, with ocrelizumab being the only approved immunomodulatory treatment shown to reduce disability progression in the Phase 3 ORATORIO trial.^7,8^

Understanding the key biological features and identifying PPMS patients at higher risk for progression is crucial for optimizing treatments. Few studies have explored predictors of clinical outcomes in PPMS. For instance, chronic white matter lesion activity can predict a 2-year disability progression,^9^ and higher baseline disability correlates with worse performance on functional tests over 2 years.^10^ Further research indicates that baseline variables, including MRI and clinical data, can predict worsening disability.^11-14^ For example, the number of enhancing T1-weighted lesions at baseline can forecast mobility decline, and a combination of clinical and radiological data can predict long-term progression.^12,13^

Additionally, transcriptomic profiles of immunocompetent cells from peripheral blood may offer predictive value of clinical outcomes.^15-19^ We previously demonstrated that PPMS patients with fast disability progression have distinct blood transcriptome profiles involving immune activation pathways.^20^

This study presents a proof-of-concept analysis demonstrating the application of machine learning framework to predict PPMS patients’ clinical outcome. By leveraging baseline transcriptional, clinical and radiological data, this approach was used to develop prognostic models capable of identifying PPMS patients likely to experience rapid disease progression, thereby highlighting those who could benefit from early treatment intervention.

## Materials and methods

### Study design

**Figure 1:**
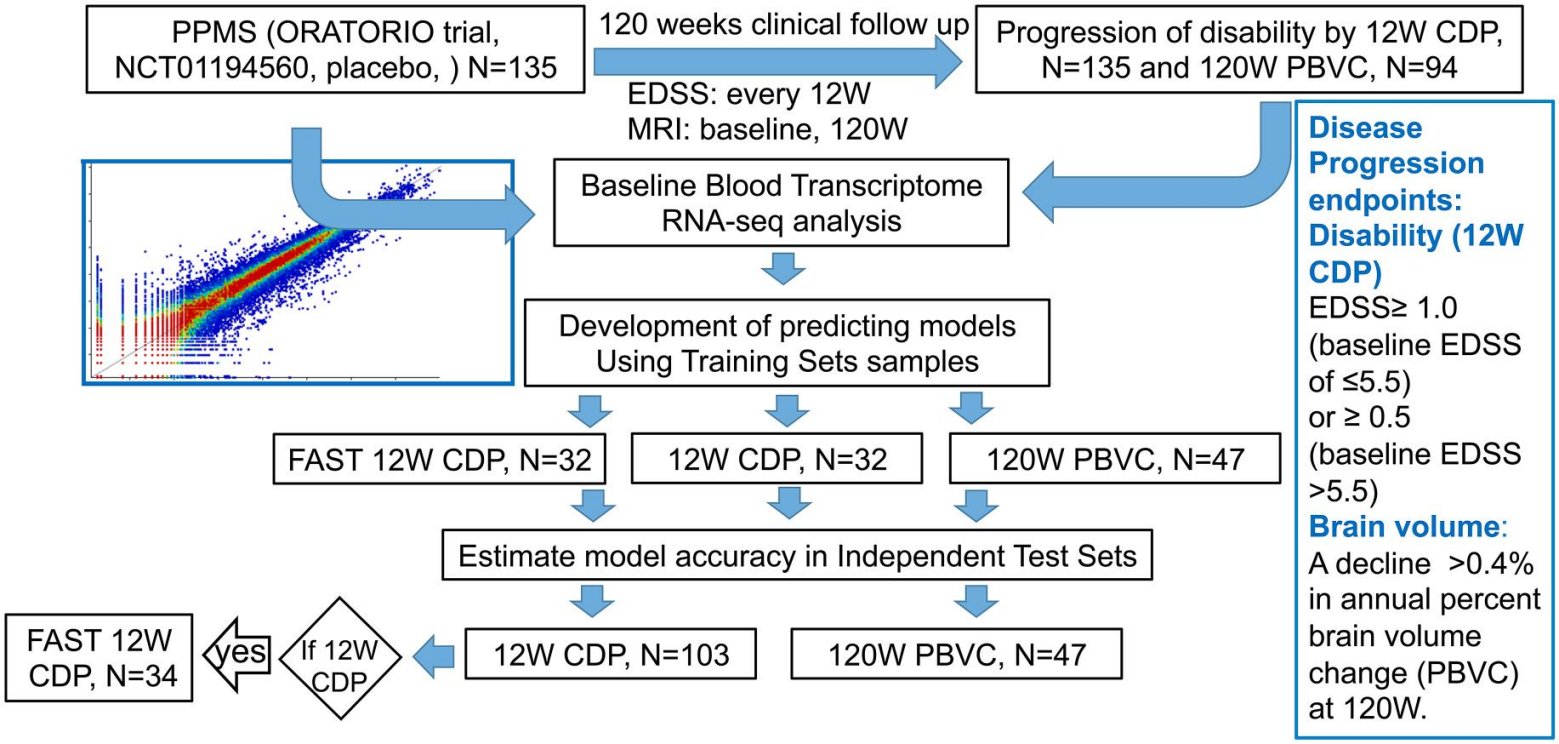
Study design.

The study design is presented in Fig. 1. Blood samples were collected from PPMS patients who participated in the placebo arm of the ORATORIO clinical trial (NCT01194560). The inclusion/exclusion criteria for participation in the study were in accordance with those of the ORATORIO trial.^7^ During the study, patients underwent clinical evaluations at baseline and regular 12-week intervals, up to 120 weeks, during which Expanded Disability Status Scale (EDSS) assessments were performed.

Brain MRI data were obtained at baseline and subsequently at weeks, 24, 48 and 120. MRI metrics included the number and volume of T2 lesions, T1 gadolinium–enhancing lesions and brain volume at baseline and 120 weeks.

### RNA-sequencing

Peripheral blood samples (2.5 ml) were collected at baseline using PAXgene vacutainers and subjected to RNA-sequencing analysis. Total RNA was purified using QiaSymphony SP, magnetic bead–based isolation method. To guarantee the construction of high-quality total RNA-seq libraries suitable for downstream analysis, the concentration and quality of RNA samples were measured using Agilent 2100 BioAnalyzer, to obtain an RNA integrity number score of ≥7.0, optical density (OD) 260/280 values between 1.8 and 2.2 and OD 260/230 values of ≥2.0.

Sequencing libraries were prepared using MARSseq. Reads were sequenced on a single lane of an Illumina NovaSeq S2. Poly-A/T stretches and Illumina adapters were trimmed from the reads using cutadapt tool,^21^ and reads shorter than 30 bp were discarded. The remaining reads were mapped onto the 3′ untranslated regions (UTR) regions (1000 bases) of the *Homo sapiens,* hg38 genome according to Refseq annotations, using the STAR alignment tool with the EndToEnd option.^22^ The outFilterMismatchNoverLmax was set at 0.05. Deduplication was carried out by flagging all reads that mapped to the same gene and shared the same unique molecular identifier. Gene counts were quantified using htseq-count,^23^ and unique molecular identifier counts were corrected for saturation by considering the expected number of unique elements in a sampling scenario without replacement. The count matrix was normalized using DESeq2,^24^ and the pipeline was run using the snakemake.^25^

### Definition of clinical progression outcomes

For the development of our prediction model, we selected two dichotomized outcomes: (i) 12-week confirmed disability progression (12W CDP, the primary outcome of the ORATORIO trial) and (ii) rate of brain volume loss. Cases in which patients reached a clinical outcome during the 120-week study period were defined as events, while those who did not reach were considered censored.

#### Disability progression

Disability progression over the 120-week study period was defined as an increase in the EDSS of at least 1.0 point from baseline, which was sustained on subsequent visits for at least 12 weeks, if the baseline score was 5.5 or less, or an increase of at least 0.5 points that was sustained for at least 12 weeks, if the baseline score was >5.5.

The analysis also included patients who withdrew from the study but met the 12W CDP criteria prior to their withdrawal (*n* = 9). Missing values were imputed as described by Montalban *et al.*^7^ To predict sustained disability progression, we designed a two-level predictive model. The first level predicted a binary outcome of progression or no progression within the 120-week study period. For patients identified by the first model as likely to experience disability progression, a second-level predictor was developed to accurately differentiate between fast and slow progressors. Patients who reached disability progression before or after the median time required to reach the clinical end-point were classified as fast or slow progressors, respectively.

#### Brain volume loss

Brain volume loss was characterized by the per cent of brain volume change (PBVC) calculated as the percentage change in brain volume from baseline to Week 120. Brain volume was dichotomized using a threshold of 0.4% annual brain volume loss, as previously described by other groups.^26,27^

### Development of predicting models

#### General principles

For prediction analysis, the entire dataset was stratified by events and baseline EDSS and split into a training set, used to build and train the model, and a test set, employed to assess the prediction performance. The predictive models were developed using two different approaches. The first approach relied solely on baseline transcriptional data, while the second approach utilized a mixed model based on the transcriptome and the relevant baseline disease clinical variables.

#### Feature selection

To eliminate redundant and irrelevant transcripts and reduce dimensionality, we focused on constructing classifiers from genes that significantly correlated with the disability progression or brain volume loss using only samples of the training set. Thereafter, classifiers were constructed using the top genes that significantly discriminated between event and censored patients (who were followed until the end of the study), with *P-*value of <0.05 after false discovery rate correction for multiple testing.

For the construction of the brain volume loss classifier, we referred to the genes that correlated with the percentage of brain volume loss over 120 weeks.

For the mixed prediction models, the baseline clinical and radiological measurements that showed significant differences between patients who reached or did not reach the relevant clinical end-point in training group were used with transcriptional features for the predictive model construction.

#### Prediction model

To select the best classification model and estimate its accuracy, we used the model selection tool implemented in Partek Genomics Suite v7.0 (Partek Inc., St. Louis, MO, USA; www.partek.com). Two methods were used to report the unbiased accuracy estimate (or correct rate): (i) one-level cross-validation using separate training set and test set samples and (ii) a two-level nested cross -validation. The estimated prediction accuracy was reported as percentage of correct classification rates (both events censored cases) when classifier applied on test set samples. Further details are presented in the Supplementary Methods.

### Statistical analysis

Continuous clinical and radiological variables are presented as mean ± standard error (SE). Student’s *t*-test was used to compare the differences in these variables between groups. Categorical variables are presented as median with a 75% interquartile range. The Mann–Whitney U-test was used to compare differences in categorical variables between groups. A *P*-value of <0.05 was considered statistically significant. For visualization and testing prediction ability of binary classification models based solely on baseline clinical and radiological parameters, the receiver operating characteristics (ROC) curve method was used. The area under the curve (AUC) and 95% confidence interval (CI) were calculated.

The probability of an event occurring versus not occurring was calculated as odds ratio (OR) with two-sided CI, where OR = (event^exposed^/non-event^exposed^)/(event^non-exposed^/non-event^non-exposed^). To convert baseline continuous parameters into binary parameters, medians were established as cut-off points for exposed and non-exposed groups. A χ^2^ test was used to compare observed results with expected.

## Results

### Study participants

From the 244 PPMS patients who received a placebo in ORATORIO clinical trial,^7^ 170 patients agreed to donate blood for the gene expression substudy (Supplementary Fig. 1 explains the patients distribution). We successfully obtained PBVC RNA-seq data from 135 patients [61 females, 74 males, mean ± SE age 44.0 ± 0.7 years, disease duration (DD) 5.86 ± 0.32 years and median baseline EDSS score 4.3 (range 3.5–6.5); Table 1].

**Table 1:** Baseline demographic and clinical parameters

End-point	Disability progression (12W CDP)	% Brain volume loss (120W PBVC)
Number of patients	*N* = 135	*N* = 94
Age, years,		
Mean ± SE	44.0 ± 0.7	43.7 ± 0.7
Median (range)	45.0 (22.0−55.0)	44.5 (22.0−55.0)
Female/male	61/74	41/53
DD, years		
Mean ± SE	5.86 ± 0.32	5.49 ± 0.33
Median (range)	5.1 (1.1−23.7)	4.8 (1.1–15.9)
EDSS, mean ± SE	4.7 ± 0.1	4.6 ± 0.1
Median (range)	4.3 (3.5−6.5)	4.0 (3.0−6.5)
EDSS ≤ 5.5, *n* (%)	89 (65.9)	66 (70.2)
EDSS > 5.5, *n* (%)	46 (34.1)	28 (29.8)
T2 lesion number		
Mean ± SE	53.1 ± 3.4	50.6 ± 3.8
Median (range)	48 (0–208)	48.5 (0−208)
T2 lesion volume, mm^3^		
Mean ± SE	12.4 ± 1.1	11.6 ± 1.4
Median (range)	8.0 (0.2−59.2)	7.1 (0.2−59.2)
Patients with T1-enhancing lesions (%)	39 (28.9)	22 (23.4)
Brain volume, mm^3^		
Mean ± SE	1464.3 ± 7.7	1473.8 ± 9.5
Median (range)	1462.0 (1263.3−1690.9)	1466.7 (1502.6−1605.5)

Over the 120-week study period, 39.3% (53/135) reached the end-point for disability progression, with an average increase of EDSS score of 1.30 ± 0.16.

For the prediction of brain volume loss, blood RNA samples from 94 PPMS patients [41 females, 53 males, mean age ± SE 43.7 ± 0.7 years and median baseline EDSS 4.0 (range, 3.0–6.5)] were included (Table 1). Sixty-seven per cent (63/94) reached the defined end-point of 0.4% annual brain volume loss. Among them, the average annual brain volume loss was −1.02 ± 0.07%, compared with a mean loss of −0.22 ± 0.05% in censored patients.

### Development of the classification model to predict disability progression

The model was trained on samples from 32 patients (training set) [mean ± SE age 43.6 ± 1.3 years, F/M ratio 10/22, median baseline EDSS 4.0 (range 3.0–6.5) and 38% events (12/32)]. The accuracy of an obtained classifier was validated using an independent subset of the remaining 103 PPMS patient samples (test set) [mean age 43.9 ± 0.8 years, F/M ratio 51/52 and median baseline EDSS 4.5 (range 3.0–6.5)]. There were no statistically significant differences between the training set and the test set in terms of age, gender and baseline EDSS (*P* > 0.3).

In the training set, we evaluated the top 10 genes that showed the most significant correlation with the rate of EDSS change (see Supplementary material) and differentiated between patients who reached the disability end-point and those who were censored. The classifier constructed from these genes consists of over-expressed *VPRBP, ZNF324, WNT10A, PHACTR4, ERGIC2* and *JAM3* and the downexpressed *SCARB1, GATB, TNFRSF10D* and *ATP5J* genes.

**Figure 2:**
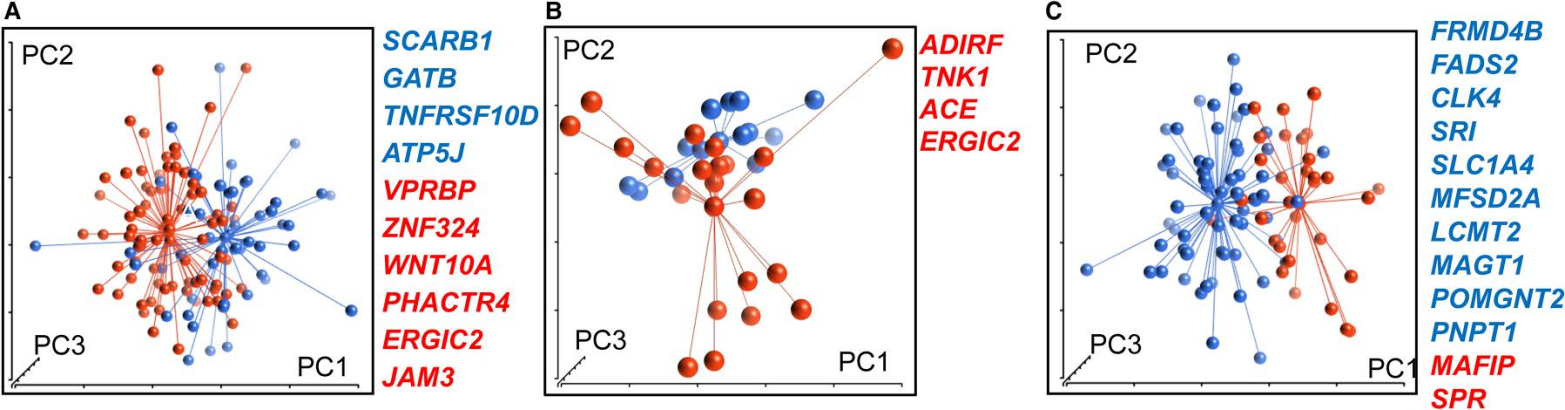
**Development of classification model.** (**A**) Principal component analysis (PCA) based on expression levels of 10 classifier genes distinguished between patients who reached disability end-point (blue dots) and censored patients (red dots), *n* = 103. (**B**) PCA based on expression levels of four classifier genes distinguished between patients with fast (blue dots) and slow disability progression (red dots), *n* = 34. (**C**) PCA based on expression levels of 12 classifier genes distinguished between patients who reached brain volume loss end-point (blue dots) and censored patients (red dots), *n* = 47. The red coloured font represents over-expressed genes and blue represents under-expressed genes. PC1, PC2 and PC3, principal components 1, 2 and 3.

We tested the classifier performance using an independent test set, comprising samples from 103 patients (39.8% events, 41/103). This yielded an estimated classification accuracy of 70.9 ± 4.5% (Table 2). Figure 2A illustrates distinct clusters of patients who reached the disability end-point and those who were censored based on the 10 genes selected for the classification model. The classifier performed comparably in patient subgroups with baseline EDSS scores of ≤5.5 points (*n* = 89) and EDSS >5.5 points (*n* = 14), achieving accuracy rates of 72 and 69%, respectively.

**Table 2:** Estimated accuracy of predictions for disability progression and brain volume loss

Prediction	Training set (*n*)	Test set (*n*)	Prediction based on	Estimated accuracy (%)	SE	AUC
Disability progression	32	103	10 genes	70.9	4.5	0.67
Fast disability progression	32	34	4 genes	74.1	5.2	0.61
Brain volume loss	47	47	12 genes	70.2	6.7	0.69
Brain volume loss	47	47	12genes + brain MRI T2 volume	74.4^a^	6.6	0.73

^a^For comparison, the estimated prediction accuracy of classifier based only on T2 volume parameters was 65.1 ± 7.2%.

### Prediction of fast and slow progressors

The cut-off definition for fast and slow disability progression was performed on samples of 53 PPMS patients [mean ± SE age 43.1 ± 0.7 years, F/M ratio 23/30, DD 5.62 ± 0.64 years and median EDSS 4.5 (range 3.0–6.5)], which reached the disability progression end-point during the study period. The median time to disability progression was 506 days: patients progressing before or after that time point were defined as fast and slow progressors, respectively. The average time to reach disability progression in fast-progressing (*n* = 29) and slow-progressing (*n* = 24) subgroups were 224 ± 29 and 773 ± 18 days, respectively.

To predict fast and slow disability progression, we trained the model on the same training set as for first-level disability predictor. We constructed an optimal classifier consisting of *ADIRF, TNK1, ACE* and *ERGIC2* genes. To evaluate the predictive accuracy of this classifier, we applied it to 34 samples from the independent test set (*n* = 103), which were predicted to reach the disability progression end-point by the first-level classifier. By using two-level nested cross-validation, within this subset of patients, the estimated classifier accuracy to predict fast disability progression was 74.1 ± 5.2% (Table 2). For this validation, patients who did not reach the disability progression end-point during 120 weeks’ follow-up were defined as slow progressors. Figure 2B illustrates distinct clusters of fast- and slow-progressing patients based on the four genes selected for the classification model.

#### Combination of baseline transcriptional, clinical and radiological data to predict confirmed disability progression

No statistically significant differences were observed in baseline age, gender, EDSS, brain volume and number and volume of T2 brain MRI lesions (T2Num, T2Vol), T2Num and T2Vol corrected for DD (T2NumCorr and T2VolCorr), calculated as T2Num/DD and T2Vol/DD. We tested all possible combinations of these parameters with gene expression–based classifier and did not observe improvement of classification accuracy to predict disability progression when compared with gene expression–based classifier alone.

### Prediction of brain volume loss

The classification model was trained on a dataset comprising 50% of the patients (47/94) and 31 (66%) events. Twelve genes correlated with the PBVC were identified as the best fitted for constructing the classifier. These included the downexpression of *CLK4, FADS2, FRMD4B, LCMT2, MAGT1, MFSD2A, PNPT1, POMGNT2, SLC1A4* and *SRI* and the over-expression of *SPR* and *MAFIP* genes. The classifier estimated accuracy to predict brain volume loss, calculated on independent test set samples from 47 PPMS patients and 32 events (68%), was 70.2 ± 6.7%. Figure 2C illustrates two distinct clusters of samples from patients who reached the brain volume loss end-point and censored based on the expression levels of the classifier genes.

#### Combining baseline transcriptional profiles and clinical parameters to predict brain volume loss

**Figure 3:**
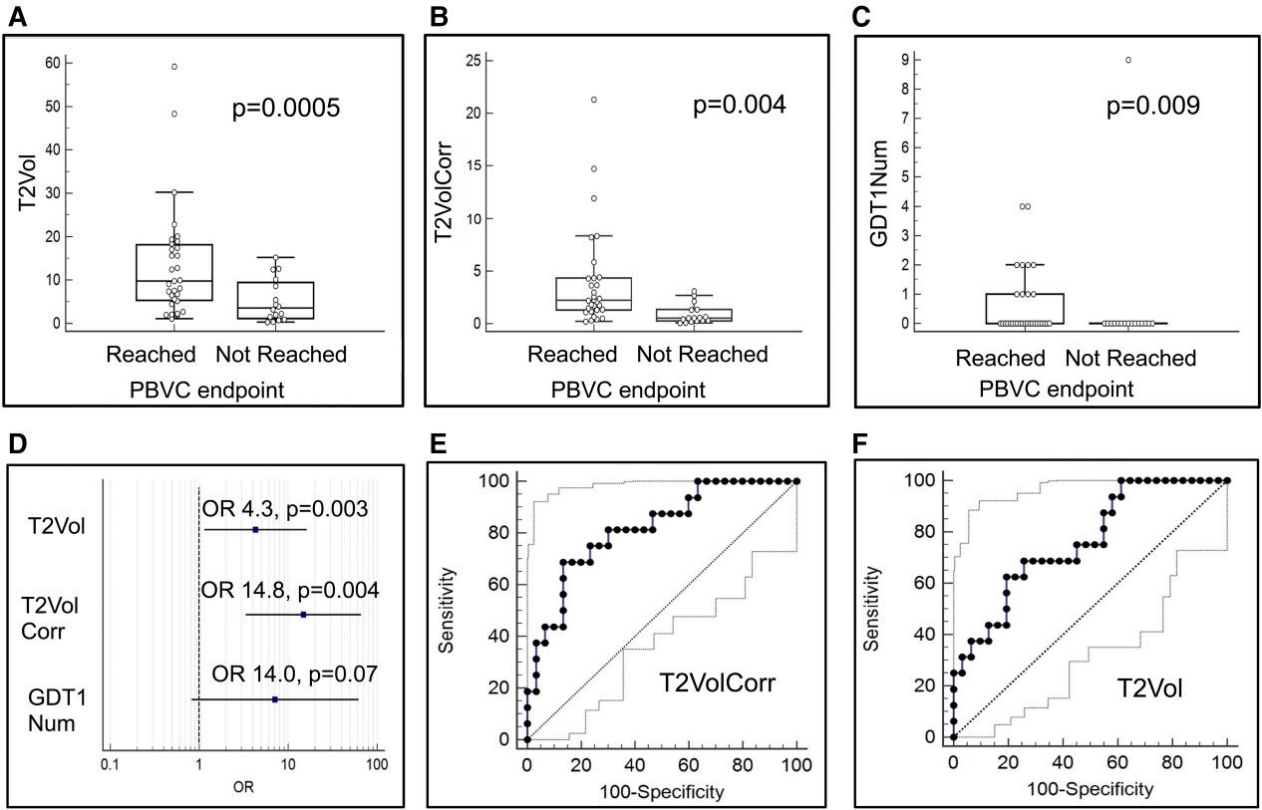
**Association between baseline radiological metrics and brain volume loss at 120 weeks of follow-up.** (**A–C**) Baseline brain MRI T2 lesions volume (T2Vol), T2 lesion volume corrected for DD (T2VolCorr) and number of gadolinium-enhancing T1 lesions (GDT1Num) were different between patients who reached brain volume loss end-point (*n* = 31) and not reached censored patients (*n* = 16; *P* = 0.0005, *t* = 3.75; *P* = 0.004, *t* = 3.03; *P* = 0.009, *t* = 2.73, respectively). (**D**) Effect of baseline radiological parameters on 120-week brain volume loss. The ORs of T2Vol, T2VolCorr and GDT1Num are demonstrated (*n* = 47). (**E** and **F**) ROC analysis of association between baseline T2VolCorr (**D**) or T2Vol (**E**) and brain volume loss at 120-week follow-up, *n* = 47. (**A**) Plot of the true positive (sensitivity) in function of false positive (100-specificity) for 0.63 mm3 for T2VolCorr and 4.37 mm3 for T2Vol cut-off points. Each point on ROC curve represents a sensitivity/specificity pair corresponding to particular decision threshold. The AUCs were 0.81 (95% CI: 0.58–0.92, *P* = 0.0001) and 0.76 (95% CI: 0.61–0.87, *P* = 0.0003) for T2VolCorr and T2Vol, respectively.

Using the same training set as above (*n* = 47), we compared the baseline clinical and radiological parameters between patients with annual brain volume loss above or below 0.4%. Patients who experienced brain volume loss >0.4% per year had significantly higher brain MRI T2 lesion volumes at baseline (13.5 ± 2.3  versus 5.23 ± 1.24 mm3; *P* = 0.009), even after adjusting T2 lesion volume for DD (3.95 ± 0.85  versus 0.89 ± 0.23 mm3; *P* = 0.007). These patients also had a higher number of T2 lesions (16.7 ± 3.3 versus 7.98 ± 2.76; *P* = 0.04) and a higher proportion of patients with T1-enhancing lesions at baseline [32.3% (10/31) versus 6.3% (1/16), *P* = 0.00001, by χ^2^ test; Fig. 3A–C].

The strong association between baseline MRI quantitation and brain volume loss is illustrated by OR for baseline T2 lesion volume [4.33, 95% CI: 1.15–16.2, *P* = 0.03 (*z* = 2.174)] and by OR for baseline T2 lesion volume corrected for DD [14.85, 95% CI: 3.35–65.9, *P* = 0.004 (*z* = 3.5); Fig. 3D].

ROC analysis of association between baseline T2 lesion volume corrected for DD (Fig. 3E) or T2 lesion volume (Fig. 3F) and 120-week brain volume loss resulted in AUCs of 0.76 (95% CI: 0.61–0.87, *P* = 0.0003) and 0.81 (95% CI: 0.58–0.92, *P* = 0.0001), respectively.

The prediction model, based solely on these brain MRI variables, yielded an estimated prediction accuracy of 65.1 ± 7.2%. However, a mixed model that combined baseline brain MRI T2 volume and T2 volume corrected for DD with the 12-gene expression classifier for brain volume loss prediction improved the estimated classifier accuracy to 74.4 ± 6.6%. Additional performance metrics can be found in the Supplementary Table 1.

Notably, out of 47 patients included in the training set, 11/47 (23%) had T1-enhancing lesions at baseline. Of them, 90% (10/11) reached the brain volume loss end-point. In contrast, only 58.3% (21/36) of the patients without T1-enhancing lesions reached the brain volume loss end-point (*P* = 0.0009, by χ^2^ test); these findings suggest that the presence of T1-enhancing lesions alone is a strong predictor for brain volume loss, with an OR of 14.0 (95% CI: 1.62–121.4, *P* = 0.008; Fig. 3D). This OR model card was presented in Supplementary Table 2. For the remaining patients without T1-enhancing lesions at baseline, we applied the 12-gene brain volume loss classifier and obtained an estimated prediction accuracy of 74% (25/34) for predicting brain volume loss (Table 2).

Taken together, using the baseline occurrence of T1-enhancing lesions in combination with the best-fitted 12-gene expression classifier for patients without enhancing lesions, the estimated accuracy to predict brain volume loss was 74.4% = (35/47).

## Discussion

Our study presents a novel machine learning approach that successfully predicted 120 weeks’ disability and brain atrophy in disease modifying drugs (DMD)-free PPMS. The innovative aspect lies in the integration of baseline high-throughput blood RNA-sequencing data with radiological variables to predict patient outcomes. By constructing classifiers based on the expression of key genes alone or in combination with brain MRI lesion counts, we achieved a significant distinction between various patient outcomes.

Prediction of clinical outcomes in placebo-treated patients plays a crucial role in analysing treatment efficacy within clinical trial treatment groups. This approach could provide robust benchmarks for evaluating treatment effects by clearly differentiating between the treatment effect and the natural course of the disease, leading to more accurate interpretation of results and better regulatory decisions. Such prediction models may allow physicians to present more realistic scenarios of disease progression without treatment, enabling patients to make more informed decisions about their treatment options. The clinical implications of predicting outcomes in placebo-treated PPMS patients extend far beyond individual clinical trials, offering invaluable insights into the natural history of PPMS and leading to improved personalized treatment and optimized healthcare resource allocation.

We tested prediction models based solely on gene expression classifiers, as well as models based on combining baseline gene expression data with baseline clinical and radiological data. Our findings indicate that baseline demographic, clinical and radiological parameters including age, gender, EDSS score, brain volume and the number and volume of brain MRI lesions did not differ significantly between patients who reached the confirmed disability progression end-point and those who did not. These results corroborate the conclusions of a systematic literature review that found baseline demographic parameters and EDSS do not predict disability progression in PPMS.^28^ Consequently, the combination of these parameters with gene expression did not enhance the accuracy of disability prediction compared with gene expression alone.

However, in the case of brain volume loss, baseline brain MRI lesions enumeration reflecting neurodegeneration in PPMS had significant ORs for the probability of reaching the brain volume loss end-point. The prediction model that combined baseline radiological parameters with gene expression data demonstrated improved prediction accuracy compared with models based on gene expression alone.

An interesting observation from our study is the role of baseline brain MRI T1 enhancing lesions in predicting brain volume loss. Of the PPMS patients involved in our biomarker substudy, 23% had enhancing brain MRI lesions at baseline, which aligns well with the 25% of patients with enhancing lesions reported in the ORATORIO clinical trial prior to ocrelizumab treatment.^7^ We found that 86.4% of patients with T1 gadolinium–enhancing lesions at baseline reached the brain volume loss end-point. Supporting our findings, Khaleeli *et al*.^11^ showed that in PPMS patients, higher baseline T1-enhancing lesions predicted a decline in mobility after 5 years. We have demonstrated that in the remaining PPMS patients without gadolinium-enhancing lesions at baseline, a high prediction rate for brain volume loss was achieved by applying the blood transcriptome–based classifier alone or in combination with baseline brain T2 lesion counts.

The predictors developed were constructed based on a relatively low number of genes; however, despite their limited number, these genes are integral to large networks underlying the molecular mechanisms and pathways associated with progression of disability (Supplementary Fig. 2). The disability classifier identified over-expression of genes such as *JAM3, ERGIC2, PHACTR4, WNT10A, ZNF324* and *VPRBP* as potential markers of disease worsening. These genes are all linked to pro-inflammatory pathways, which contribute to neuroinflammation, demyelination and axonal damage. *JAM3* encodes a protein that regulates leucocyte transmigration and angiogenesis. Over-expression of *JAM3* has been associated with increased vascular permeability and immune cell infiltration into the central nervous system.^29,30^ The *ERGIC2* gene, encoding a protein involved in protein trafficking and secretion, has been found to increase the production of pro-inflammatory cytokines and chemokines by enhancing lipopolysaccharide-mediated tumour necrosis factor-α production in monocytes.^31,32^ Similarly, over-expression of *ZNF324* gene has been associated with increased cell proliferation and the expression of pro-inflammatory cytokines and chemokines.^33^ Viral protein R binding protein (VPRBP), a component of the CUL4A-RBX1-DDB1-DCAF1/VPRBP complex crucial for B-cell development, is implicated in limiting error-prone repair during V(D)J recombination,^34^ a significant process, given the targeting of B-cells by ocrelizumab. The inclusion of B-cell–related genes in the disability progression classifier could explain the therapeutic efficacy of Ocrelizumab, which depletes B-cells, thereby confirming its therapeutic role in PPMS. *WNT10A*, a member of the large wingless-related integration site (WNT) gene family known for encoding signalling proteins, contributes to chronic neuroinflammation through the modulation of NFkB pathway. WNT signalling machinery proteins regulate T-cell transcription factors 1, 3 and 4 and the lymphoid enhancer factor transcription factor,^35^ influencing the balance between effector and memory cells.^36^ Last, PHACTR4, a member of the phosphatase and actin regulator family, and its homologue PHACTR1 have been demonstrated to mediate monocyte adhesion by activating nuclear factor kappa-light-chain-enhancer of activated B cells (NFκB)-dependent intercellular adhesion via intercellular adhesion molecule 1 (ICAM1) and vascular cell adhesion molecule 1 (VCAM1).^37^

The under-expressed genes within the disability prediction classifier act as negative regulators of inflammation. The *SCARB1* gene, encodes a protein that is part of the scavenger receptor B-cell surface protein family, predominantly expressed by macrophages and monocytes. It was shown that *SCARB1-*deficient mice exhibit a 3- to 4-fold increase in B and T lymphocyte activation, a shift towards pro-inflammatory Th1/Th2 ratio and the induction of systemic autoimmune diseases.^38^ The *TNFRSF10B* gene, encoding the TNF-related apoptosis inducing ligand (TRAIL) receptor that triggers apoptosis, was reported to be associated with reduced apoptosis in multiple sclerosis patients and thereby prolong the survival of autoreactive immune cells.^39,40^ In accordance, *TRAIL-*deficient mice are highly susceptible to experimental autoimmune encephalomyelitis, while *in vivo* administration of embryonic stem cell–derived dendritic cells expressing TRAIL decreased the severity of the disease.^41^

The functional ontology analysis of the four genes included in the classifier predicting fast or slow disability progression highlights their association with inflammatory pathways. *ADIRF* gene, an adipogenesis regulatory factor, which is upregulated in naïve CD4 and CD8 T cells,^42^ has been linked to inflammatory mechanisms in Alzheimer’s disease.^43^ Similarly, over-expression of *TNK1*, which encodes 38 negative kinase 1 protein, has been reported to activate STAT3, p65, interleukin-6 and tumour necrosis factor cytokine production,^44^ in addition to enhancing focal adhesion mechanisms.^45^ The *ACE* gene, encoding the serum angiotensin-converting enzyme, has been associated with an increased risk of developing multiple sclerosis due to polymorphism.^46^ Moreover, elevated serum levels of ACE in multiple sclerosis patients were reported to correlate with clinical and radiological markers of disease severity.^47^ It has been demonstrated that ACE inhibitors ameliorate clinical signs of experimental autoimmune encephalomyelitis in mice and reduced lymphocyte infiltration into the CNS.^48^ The fourth gene, *ERGIC2*, has a role in transport between the endoplasmic reticulum and the Golgi apparatus. According to the Human Protein Atlas, ERGIC2 is detected in all immune cells, with higher expression levels in B-cell plasma blasts and neutrophils and is also a part of the oligodendrocytes-related cluster genes.^42^ Notably, the inclusion of the *ERGIC2* gene in the first-level disability classifier confirms its predictive power.

The transcripts included in the brain volume loss classifier could explain a more aggressive ongoing inflammatory and neurodegenerative processes contributing to brain volume loss. Over-expression of *MAFIP*, known to inhibit the activation of the NFkB pathway,^49^ alongside the effects of NFkB suppression in the nervous system,^50^ could enhance neurodegeneration and diminish neuroprotection. The *SPR* gene that encodes sepiapterin reductase is known to catalyse the final step in the biosynthetic pathway of tetrahydrobiopterin [BH(4)] enzyme, involved in dopamine synthesis. Activation of BH4 can stimulate inflammatory response,^51^ with elevated levels of BH4 pathway metabolites augmenting CD4^+^ and CD8^+^ T-cell responses, while its suppression abrogates T-cell–mediated immunity.^52^ Increased SPR expression was demonstrated in the brain Parkinson's disease patients.^53^ Interestingly, neopterin, one of the metabolites of BH4 synthesis, is a well-known marker of immune cells activity in neurodegenerative diseases, including multiple sclerosis.^54^

The *FRMD4B* (FERM domain containing 4B) gene, associated with cognitive function determinants,^55^ is also linked to brain volume, both in healthy populations and multiple sclerosis patients.^56,57^ Additionally, the *FRMD4B* gene was implicated in the myelination network in late-onset of Alzheimer disease.^58^ The *MAGT1* gene encodes a protein that regulates intracellular Mg2 and protein glycosylation. Deficiencies in *MAGT1* lead to significant glycosylation defects and altered expression of genes involved in CD28 immune mechanism,^59^ with *MAGT1* knock-out in mice resulting in increased frequency of CD19^+^ B lymphocytes.^60^ Glycoprotein quantification has been recently applied in the diagnosis and prognosis neurodegenerative diseases.^61^

The *MFSD2A* gene, expressed in oligodendrocyte precursor cells, is essential for lysophosphatidylcholine uptake during myelination.^62^ MFSD2A-deficient mice are characterized by neuronal cell loss in the hippocampus and cerebellum, as well as cognitive deficits, severe anxiety and microcephaly.^63^ The *SLC1A4* gene, a sodium-dependent neutral amino acid transporter, is highly expressed in many tissues, including the brain, primarily in astrocytes. It plays a key role in neuronal differentiation and development, maintaining neurotransmitter homeostasis and N-methyl-D-aspartate (NMDA) neurotransmission, through the regulation of L- and D-serine. SLC1A4 mutations are associated with a rare autosomal recessive neurodevelopmental disorder characterized by spastic tetraplegia, thin corpus callosum and progressive microcephaly,^64^ associated with delayed myelination.^65^ Downregulation of SLC1A4 protein levels in astrocytes from brain white matter has been associated with acute multiple sclerosis.^66^

One of the limitations of our study is the relatively small dataset. To address this, we have undertaken extensive efforts to ensure reliable internal and external validity. These efforts include incorporating active learning techniques and sample balancing strategies designed to enhance model performance while minimizing overfitting. Additionally, we employed resampling approaches to provide reliable estimates of model performance, particularly for small datasets. The statistically significant accuracy of the predictors on randomly chosen test samples for external validity suggests that our results are likely valid and generalizable to the target population.

Both predicted clinical outcomes disability progression and brain volume loss are continuous measurements rather than categorical. However, we developed binary classification prediction models, which required defining cut-off levels for clinical end-points. For instance, we used a 0.4% annual PBVC cut-off, although different cut points could be chosen. In establishing the cut-off point for our study, we considered the age range of participants and referred to previous statistical studies,^67,68^ which demonstrate that a 0.4% annualized brain volume loss associated with healthy aging could be used as a cut-off to distinguish pathological changes in multiple sclerosis patients (with a similar age range of 18–63 years as in our cohort). An additional limitation related to, a restricted time window, with the clinical outcome end-point being binary-labeled if reached within 120 weeks of follow-up. Future investigations that consider the prediction of time-to-event rather than binary clinical outcomes may improve the models.

## Conclusion

Our study provides the first proof-of-concept to accurately predict disability progression and brain volume loss in PPMS. It is of importance to further investigate these classifiers in a population of treated PPMS patients, to evaluate the impact of immunomodulatory treatment on the prediction outcomes. The identified classifiers could help understand why certain patients progress faster or respond to a particular medication by highlighting the underlying genetic differences.

## Supplementary Material

fcae427_Supplementary_Data

## Data Availability

The data that support the findings of this study are available from the corresponding author, upon reasonable request.
