# Supplementary material for: Machine learning–based prediction of disease progression in primary progressive multiple sclerosis
Source: Brain Commun. 2025 Jan 8;7(1):fcae427. doi: 10.1093/braincomms/fcae427 (PMC11707605; doi:10.1093/braincomms/fcae427)
Supplement: fcae427_Supplementary_Data [file fcae427_supplementary_data.docx]

**Supplementary Data**

**Prediction Analysis.**

For the prediction analysis, aimed to select the best classification model and estimate accuracy of the chosen model, we utilized the model selection tool implemented in Partek Genomics Suite v7.0 (Partek, Inc., St. Louis, MO, USA) (www.partek.com).

Two approaches were employed to report unbiased accuracy estimates:

(a) 1-level of cross-validation using independent Training Set and Test Set samples, and (b) 2-level nested cross-validation.

In 1-level cross-validation, applied to Training Set samples for the development of disability progression and brain volume loss predictors, the Training Set samples were initially divided into 10 random partitions. In each iteration, 10% of samples were held out for testing, while the remaining 90% were used to fit the model parameters. The best predictive model was then deployed and evaluated on completely independent Test Set samples.

Two-level nested cross-validation was applied to the Test Set to assess the classification accuracy of second-level predictors for Fast or Slow disability progression. This method involved N outer and N inner cross-validation data partitions. In each outer cross-validation, 1/N-th of the Test Set samples were held out as test samples, and the remaining samples were used in an inner N-fold cross-validation. This inner N-fold cross-validation was repeated N times to determine optimal classifier parameters. The model with superior performance on the inner cross-validation subset was applied to the samples held out in the outer cross-validation. This process was repeated N times, with the inner cross-validation optimizing the model, and the outer cross-validation assessing overall accuracy estimates for the classifier.

For all models, the classification algorithms tested included K-Nearest Neighbor, Nearest Centroid, Discriminant Analysis, Support Vector Machine, Partial Least Squares, Diagonal Discriminant Analysis, Random Forest, and Logistic Regression. Variable parameters in each classification model setting were considered as different classification models.

**Prediction of disability progression.**

To predict sustained disability progression, a two-level predictive model was designed. The first level predicted whether progression would occur within the 120-week study period, providing a binary outcome. For patients identified as likely to experience disability progression by the first model, a second-level predictor was developed to differentiate between fast and slow progression rates.

**First level disability predictor.**

135 samples (53 events, 39.3%) that passed quality control criteria during RNA-Seq were used for development of classification algorithm to predict disability progression as demonstrated by 12 week confirmed disability progression (12W CDP). This included patients that completed 120 weeks follow-up (reached or not reached 12W CDP), as well as patients that withdrew from study but fulfilled the 12W CDP requirements before withdrawal. The patients were stratified for baseline EDSS, disability events and time to disability event, and 32 patients were designated as the Training Set and the remaining 103 patients served as the Test Set. Patients with imputed 12W CDP missing values were not included in the Training Set.

**Feature selection.**

Feature selection was performed using Training Set samples to eliminate redundant and irrelevant transcripts and reduce dimensionality. Exclusively utilizing samples within the Training Set group, our focus was on constructing classifiers from genes that exhibited a significant correlation with the rate of corrected EDSS change. The corrected EDSS changes were computed, taking into consideration that the definition of disability progression in ORATORIO study^7^ was dependent on baseline EDSS. We linearized the EDSS change data by applying a 2-fold multiplication to each EDSS change in patients with a baseline EDSS greater than 5.5. Subsequently, the rate of corrected EDSS change was determined using the following formula:

*Rate of corrected EDSS change = corrected EDSS change/time to disability endpoint.*

Following this, classifiers were constructed using the top genes that significantly discriminated (by T-test) between that reached 12W CDP and censored patients who do not experience such event (followed to the end of the study), with a p-value less than 0.05 after False Discovery Rate (FDR) correction for multiple testing.

**Second level disability predictor**. The categorization of patients into 'Fast' and 'Slow' progression groups was determined based on the median time taken to reach disability progression. Patients achieving disability progression faster than the median time were classified as Fast progressors, while those taking longer than the median time were classified as Slow progressors. To predict Fast and Slow disability progression, we trained the model on the same Training Set as for the first-level disability predictor (n=32). An optimal classifier was constructed based on genes significantly correlated with the time to reaching the endpoint of disability progression and that significantly discriminated (by T-test) between Fast and Slow progressors. Subsequently, the obtained second-level predictor was tested using two-level nested cross-validation on the 34 from the Test Set of 103 patients predicted to have a disability progression endpoint by the first-level predictor.

The two-level nested cross-validation method was executed using 5 outer and 5 inner cross-validation data partitions. In each outer cross-validation, 1/5-th of the 34 Test Set samples (n=6 or n=7) were held out as test samples, and the remaining 28 or 27 samples were used in an inner 5-fold cross-validation. In each iteration of the inner cross-validation, 1/5-th of these samples (n=5 or n=6) were held out, and the process was repeated 5 times to determine optimal classifier parameters. The model with better performance on the inner cross-validation subset was applied to the samples held out in the outer cross-validation. This process was repeated 5 times.

**The Brain Volume Loss Predictor.**

Brain volume loss was defined as the percentage of brain volume change (PBVC) and was calculated as the adjusted percentage change in brain volume from baseline to week 120. To convert brain volume loss into a binary parameter, a cutoff point was established a using a threshold of 0.4% annual brain volume loss. This determination was based on the consideration that individuals over the age of 40 typically experience an annual brain volume decline of 0.4%). Using 50% of the available data as a Training Set (n=47), we evaluated genes significantly correlated with the percentage of brain volume loss and constructed a classifier using a 1-level cross-validation algorithm. An additional mixed model based on combination of baseline clinical and radiological parameters with blood RNA-Seq data was also applied. Subsequently, hold-out validation was performed on completely independent 47 samples of the Test Set

**Supplementary Table 1. Brain volume loss prediction based on combination of baseline blood gene expression and radiological parameters (n=43). ***

| **Positive Outcome** | Reaching brain volume loss endpoint |
| --- | --- |
| **Negative Outcome** | Outcome(s) other than positive |
| **Actual Positive (P):** | 30 |
| **Actual Negative (N):** | 13 |
| **Predictived Positive (P'):** | 27 |
| **Predictived Negative (N'):** | 16 |
| **True Positive (TP):** | 23 |
| **False Positive (FP):** | 4 |
| **False Negative (FN):** | 7 |
| **True Negative (TN):** | 9 |
| **Sensitivity (TP/(TP+FN)):** | 0.7667 |
| **Specificity (TN/(FP+TN)):** | 0.6923 |
| **Positive Predictive Value (TP/(TP+FP)):** | 0.8519 |
| **Negative Predictive Value (TN/(FN+TN)):** | 0.5625 |
| **Matthews Correlation Coefficient ((TP*TN-FP*FN)/sqrt(P*N*P'*N')):** | 0.4361 |
| **Area Under Curve (((TP/(TP+FN)) + (TN/FP+TN))*0.5):** | 0.7295 |

*In this Test Set (n=47) for PBCV prediction 4 patients didn’t have associated radiological parameters.

**Supplementary Table 2. Odds baseline brain MRI T1 gadolinium enhancing lesions in reaching brain volume loss endpoint during 120 weeks follow up.**

| **Odds ratio model card** | **Training Set (n=47)** | **All data (n=94)** |
| --- | --- | --- |
| Events in exposed group | 10 | 19 |
| Non-events in exposed group | 1 | 3 |
| Events in non-exposed group | 15 | 38 |
| Non-events in non-exposed group | 21 | 34 |
| Odds ratio | 14 | 5.66 |
| 95% CI | 1.65-121.40 | 1.54-20.85 |
| P | 0.008 | 0.004 |
| Z | 2.39 | 2.60 |

Exposed - defined patients with brain MRI T1 gadolinium enhancing lesions at baseline. Events - defined patients reached brain volume loss endpoint at 120 weeks follow up

**Supplementary Figure 1. Study design with patients dispositions.**

* - 94/135 patients had registered PBVC; ** - Training Set for Second level fast 12W CDP prediction the same as the Training Set for the First Level 12W CDP prediction; *** - Second Level Fast 12W CDP classifier was tested on 34 from 103 patients that were predicted to reach disability endpoint by First Level 12W CDP classifier.

**
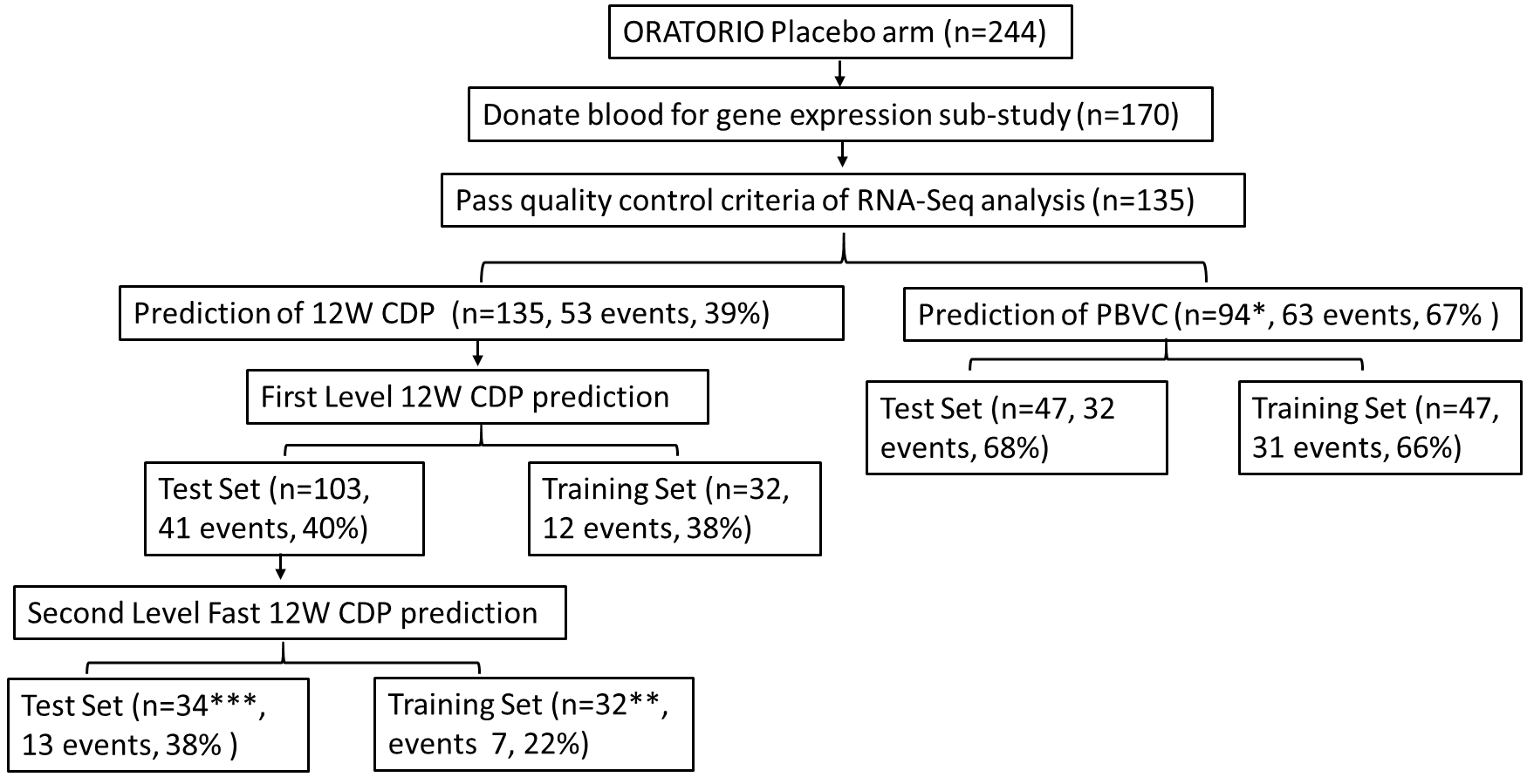
**

**Supplementary Figure 2. The regulatory network of 10-genes classifier for prediction 2 years disability progression in PPMS.**

The over-expressed genes represented by red color, down-expressed – by purple color. Genes with blue color represented predicted inhibition and with orange color – predicted activation. Orange lines represented activation effects, blue lines – inhibition effects, yellow lines – inconsistent findings with state of downstream genes, gray color represented not predicted connections. Solid lines represented direct interactions, dotted lines – indirect.

**
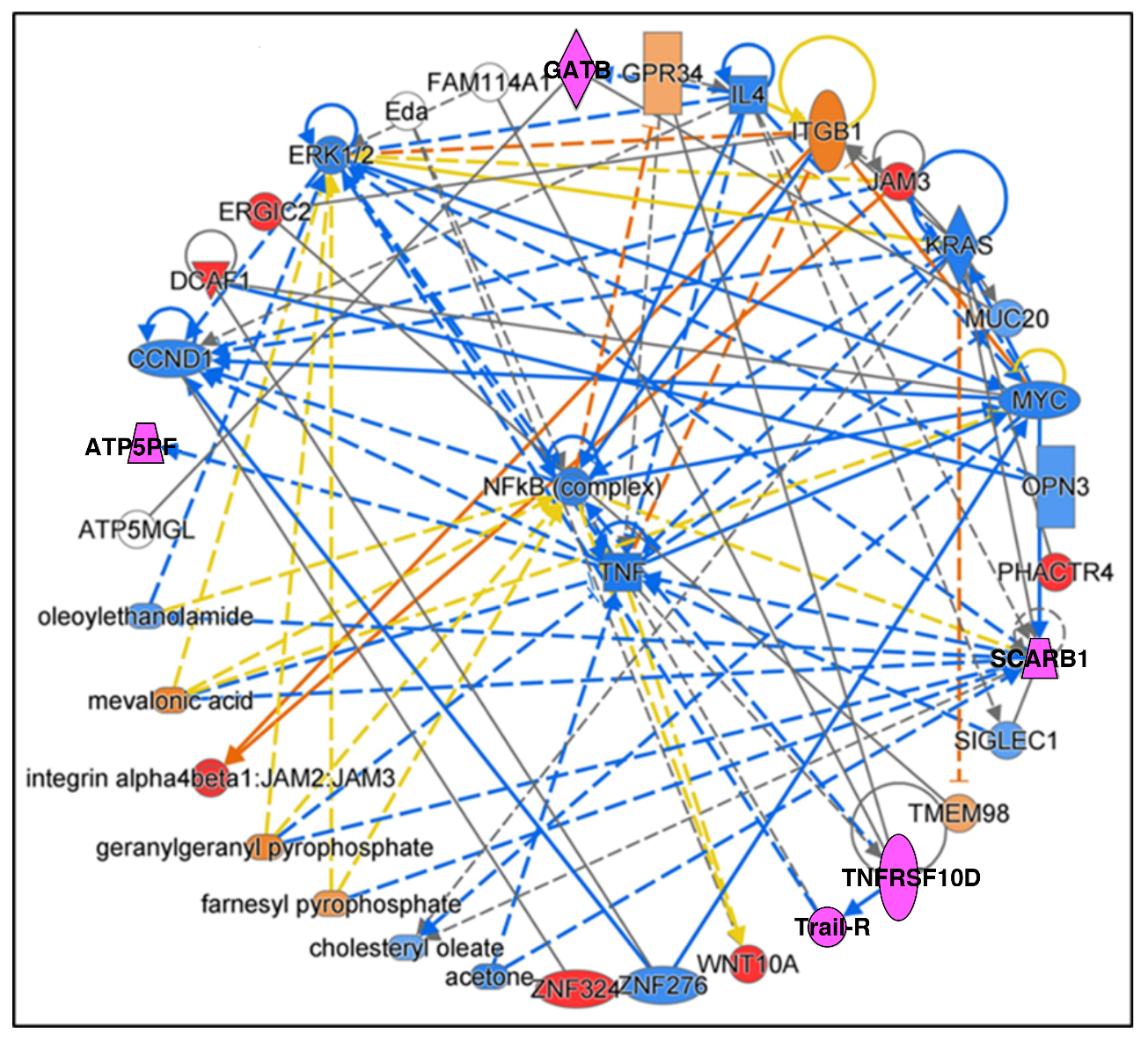
**
